# The Rare, the Best: Spread of Antimalarial-Resistant Plasmodium falciparum Parasites by *Anopheles* Mosquito Vectors

**DOI:** 10.1128/Spectrum.00852-21

**Published:** 2021-10-20

**Authors:** Antoine Berry, Sandie Menard, Sandrine E. Nsango, Luc Abate, Didier Concordet, Majoline Tchioffo Tsapi, Xavier Iriart, Parfait H. Awono-Ambéné, Benjamin Roche, Isabelle Morlais

**Affiliations:** a Institut Toulousain des Maladies Infectieuses et Inflammatoires (Infinity), Université Toulouse, CNRS UMR5051, INSERM UMR1291, UPS, Toulouse, France; b Centre Hospitalier Universitaire de Toulouse, Hôpital Purpan, Service de Parasitologie-Mycologie, Toulouse, France; c Department of Biological Sciences, Faculté de Médecine et des Sciences Pharmaceutiques, Université de Douala, Douala, Cameroon; d Malaria Research Unit, Centre Pasteur du Cameroun, Yaoundé, Cameroon; e MIVEGEC, IRD, CNRS, Université de Montpellier, Montpellier, France; f INTHERES, Université de Toulouse, INRA, ENVT, Toulouse, France; g Laboratoire d’Entomologie Médicale, Organisation de Coordination pour la Lutte contre les Endémies en Afrique Centrale, Yaoundé, Cameroon; h International Center for Mathematical and Computational Modeling of Complex Systems (UMI IRD/UPMC UMMISCO), Bondy, France; i Departamento de Etología, Fauna Silvestre y Animales de Laboratorio, Facultad de Medicina Veterinaria y Zootecnia, Universidad Nacional Autónoma de México (UNAM), Ciudad de México, México; Weill Cornell Medicine

**Keywords:** malaria, *Anopheles*, transmission, *Plasmodium falciparum*, resistance, antimalarials, genetic diversity, mosquito

## Abstract

The emergence of resistance to antimalarials has prompted the steady switch to novel therapies for decades. Withdrawal of antimalarials, such as chloroquine in sub-Saharan Africa in the late 1990s, led to rapid declines in the prevalence of resistance markers after a few years, raising the possibility of reintroducing them for malaria treatment. Here, we provide evidence that the mosquito vector plays a crucial role in maintaining parasite genetic diversity. We followed the transmission dynamics of Plasmodium falciparum parasites through its vector in natural infections from gametocytes contained in the blood of asymptomatic volunteers until sporozoites subsequently developed in the mosquito salivary glands. We did not find any selection of the mutant or wild-type *pfcrt* 76 allele during development in the *Anopheles* mosquito vector. However, microsatellite genotyping indicated that minority genotypes were favored during transmission through the mosquito. The analysis of changes in the proportions of mutant and wild-type *pfcrt* 76 alleles showed that, regardless of the genotype, the less-represented allele in the gametocyte population was more abundant in mosquito salivary glands, indicating a selective advantage of the minority allele in the vector. Selection of minority genotypes in the vector would explain the persistence of drug-resistant alleles in the absence of drug pressure in areas with high malaria endemicity and high genetic diversity. Our results may have important epidemiological implications, as they predict the rapid re-emergence and spread of resistant genotypes if antimalarials that had previously selected resistant parasites are reintroduced for malaria prevention or treatment.

**IMPORTANCE** Drug selection pressure in malaria patients is the cause of the emergence of resistant parasites. Resistance imposes a fitness cost for parasites in untreated infections, so withdrawal of the drug leads to the return of susceptible parasites. Little is known about the role of the malaria vector in this phenomenon. In an experimental study conducted in Cameroon, an area of high malaria transmission, we showed that the vector did not favor the parasites based on sensitivity or resistance criteria, but it did favor the selection of minority clones. This finding shows that the vector increases the diversity of plasmodial populations and could play an important role in *falciparum* malaria epidemiology by maintaining resistant clones despite the absence of therapeutic pressure.

## INTRODUCTION

Antimalarial drug resistance represents one of the main obstacles for the control of malaria (1, 2). Indeed, Plasmodium falciparum has an outstanding ability to develop resistance to numerous drug regimens, including the most efficient ones, like artemisinins. Drug resistance to P. falciparum is associated with specific mutations or gene amplification that confer a fitness advantage to mutant parasites when exposed to drugs. In contrast, drug resistance imposes a fitness cost in untreated infections, and withdrawal of the drug leads to the return of sensitive parasites (3–6). Chloroquine (CQ) resistance (CQR) is related to point mutations in the P. falciparum chloroquine resistance transporter (*pfcrt*) gene, and the *pfcrt* K76T mutation (encoding a change of K to T at position 76) is the best-known CQR marker (7).

Malaria infections are complex under natural conditions and generally harbor multiple genotypes of parasites (8, 9). Several lines of evidence suggest that genetic diversity within infections correlates with transmission intensity (10, 11), but whether the genetic composition influences the transmission dynamics remains elusive. Transmission of malaria parasites depends on the successful development of the sexual stages, the gametocytes, within the mosquito vectors. Parasite population structure and dynamics in the insect vector are complex and influenced by numerous factors from human or vector hosts (12–17). We have previously shown that mixed-genotype infections in humans lead to lower parasite burdens in mosquitoes, supporting the existence of competitive interactions between coinfecting genotypes (17, 18). However, the importance of mosquitoes in the transmission of drug-resistant parasites in naturally acquired multiple-genotype infections is still poorly understood.

In this study, we investigated the genetic diversity of P. falciparum parasites in both gametocytes isolated from the blood of asymptomatic donors and salivary glands dissected from mosquitoes experimentally fed with blood from the same gametocyte donors. Samples were genotyped at the *pfcrt* 76 codon and at 7 microsatellite loci. We then assessed the role of mosquitoes in the spread of *Plasmodium* resistance to antimalarial drugs within the population. Our results shed light on the key role of the mosquito vector in shaping the antimalarial-resistant parasite population.

## RESULTS

### Characteristics of the infections.

P. falciparum gametocyte-containing blood from 67 naturally infected carriers was used to isolate gametocyte samples and to perform experimental feedings. The median gametocyte density of blood donors was 53 parasites/μl (minimum = 11; maximum = 2,304) (Table S1 in the supplemental material). A total of 723 mosquito salivary gland samples were P. falciparum positive.

### No selection of the mutant or wild-type *pfcrt* 76 alleles by the *Anopheles* mosquito vector.

**FIG 1:**
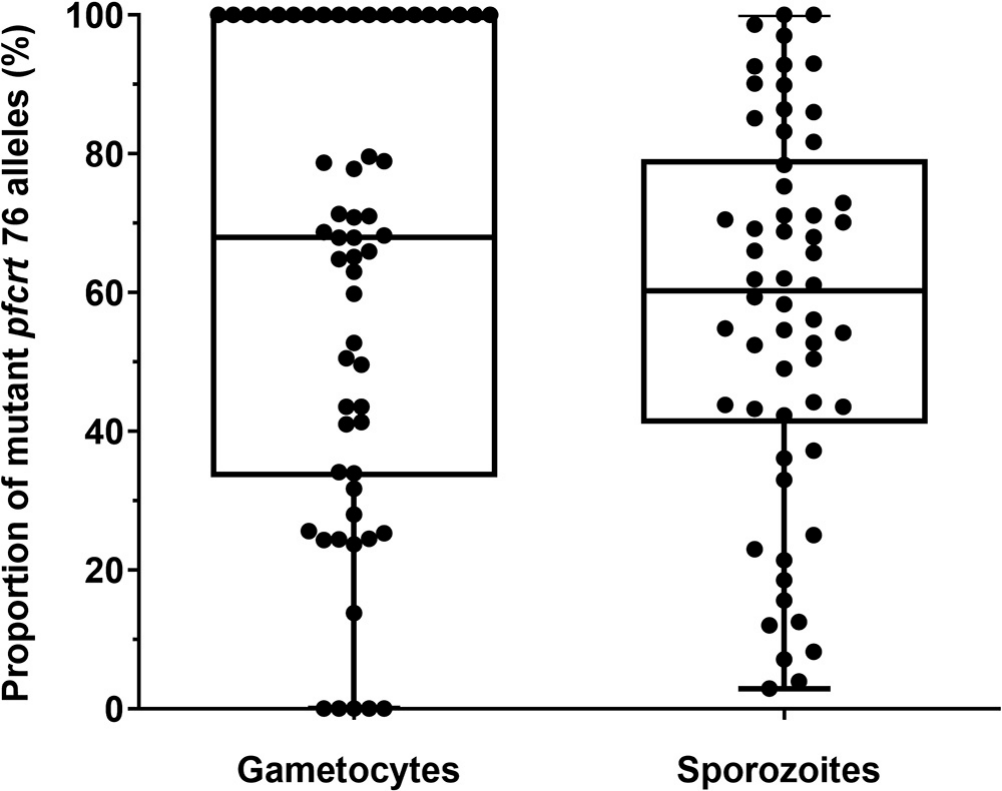
Box plot of mutant *pfcrt* 76 alleles in gametocyte and sporozoite samples. The dots represent the observed proportions of mutant *pfcrt* 76 alleles in the gametocyte samples and the observed average proportions of mutant *pfcrt* 76 alleles in the sporozoite samples. Box plots represent the median, 25th, and 75th percentiles and the minimum and maximum.

We excluded from the analysis all blood donors carrying a single genotype, either the mutant or the wild-type *pfcrt* 76 allele, in the gametocyte samples and all the paired sporozoite samples. Thus, 57 gametocyte samples and 643 sporozoite (salivary gland) samples, with a mean of 11 (minimum = 1; maximum = 24) mosquitoes per blood donor, were analyzed for *pfcrt* 76 alleles (Table S1). No selection of the mutant (or wild-type) allele was observed, as the median of the proportion of mutants (67.9%; interquartile range [IQR], 33.4% to 100.0%) in gametocytes was not different from that of paired sporozoite samples (mutant allele: 60.2%; IQR, 41.3% to 79.2%; Wilcoxon signed-rank test, *P = *0.268) (Fig. 1). By symmetry, the same observation was made for the wild-type allele (Fig. S3).

### The *pfcrt* 76 allele had an advantage for transmission from humans to the vector when present at very low frequencies.

**FIG 2:**
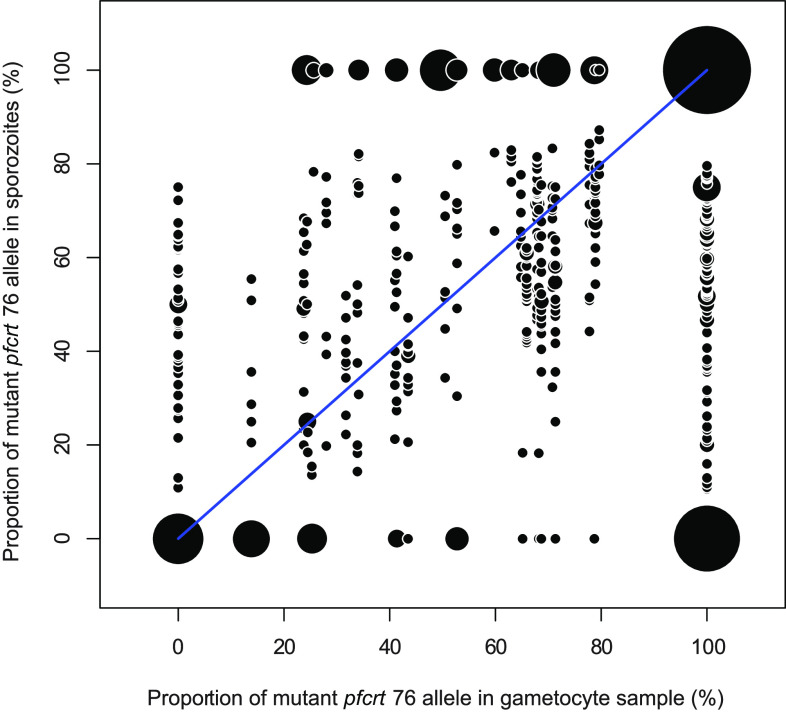
Dispersion of the proportions of mutant *pfcrt* 76 alleles in gametocyte samples and in paired sporozoite samples. The dots represent the observed proportion of mutant *pfcrt* 76 alleles in each sporozoite sample against the observed proportion of mutant *pfcrt* 76 alleles in the paired gametocyte sample. The blue line represents the expected and theoretical distribution of dots if the vector has no influence in the transmission of *pfcrt* 76 alleles. The dot size is weighted by the number of samples.

**FIG 3:**
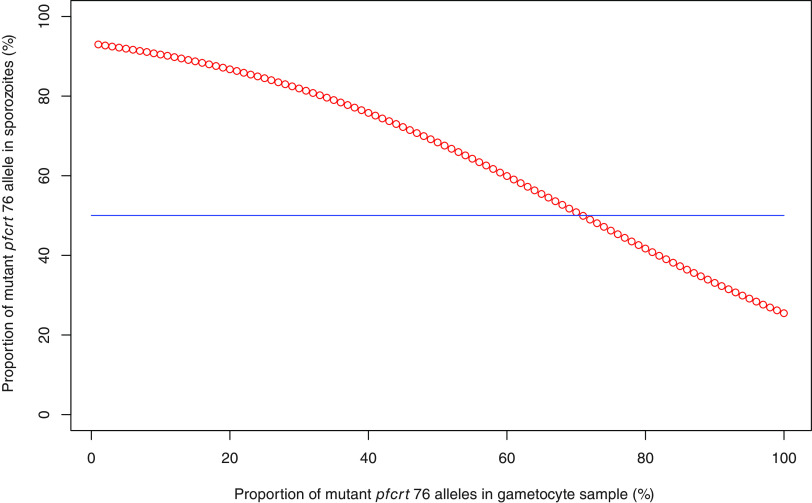
Logistic regression of the probability of a higher proportion of mutant *pfcrt* 76 alleles in sporozoite samples as a function of the proportion of mutant *pfcrt* 76 alleles in gametocyte samples. The proportion of mutant *pfcrt* 76 alleles in sporozoite samples was compared to that in the paired gametocyte samples. The probability of having a higher proportion of mutant *pfcrt* 76 alleles in sporozoite samples was calculated for each gametocyte sample using a logistic regression; the donor effect was taken into account. This probability (expressed as a percentage) as a function of the proportion of mutant *pfcrt* 76 alleles in gametocyte samples was plotted (red line). The blue line represents the expected probability if the variation of the proportion of mutant *pfcrt* 76 alleles in sporozoite samples is randomly distributed (50% of a higher proportion and 50% of a lower proportion). By symmetry, the result was identical for the wild-type allele (see Fig. S3).

For each parasite donor, the fraction of the *pfcrt* 76 allele obtained for the sporozoite samples was plotted against the one obtained for the corresponding gametocyte samples (Fig. 2 and Fig. S4). The *pfcrt* 76 proportion in the sporozoite samples is expected to be similar to the one in the gametocyte sample in the absence of selection through the mosquito vector (blue line in Fig. 2 and Fig. S4). We tested this hypothesis using a logistic regression where the proportion of the mutant *pfcrt* 76 allele in the gametocyte sample was compared to that of the paired sporozoite samples. The probability of observing a higher proportion of the *pfcrt* 76 alleles in sporozoite samples than in gametocyte samples (red line in Fig. 3) was significantly different (*P = *5.29e−05) from the expected 50% probability (blue line in Fig. 3) for the mutant *pfcrt* 76 allele. As the proportion of mutant *pfcrt* 76 alleles decreased in gametocyte samples, the probability of having a higher proportion of this mutant allele in sporozoite samples increased, while as the proportion of mutant *pfcrt* 76 alleles increased in gametocyte samples, the probability of having a lower proportion of this mutant allele in sporozoite samples increased. By symmetry and logically, the same observation was made for the wild-type allele (Fig. S5). These results suggest that the minority alleles, whether mutant or wild type, had a selective advantage within a parasite population in the transmission from humans to the vector.

**FIG 4:**
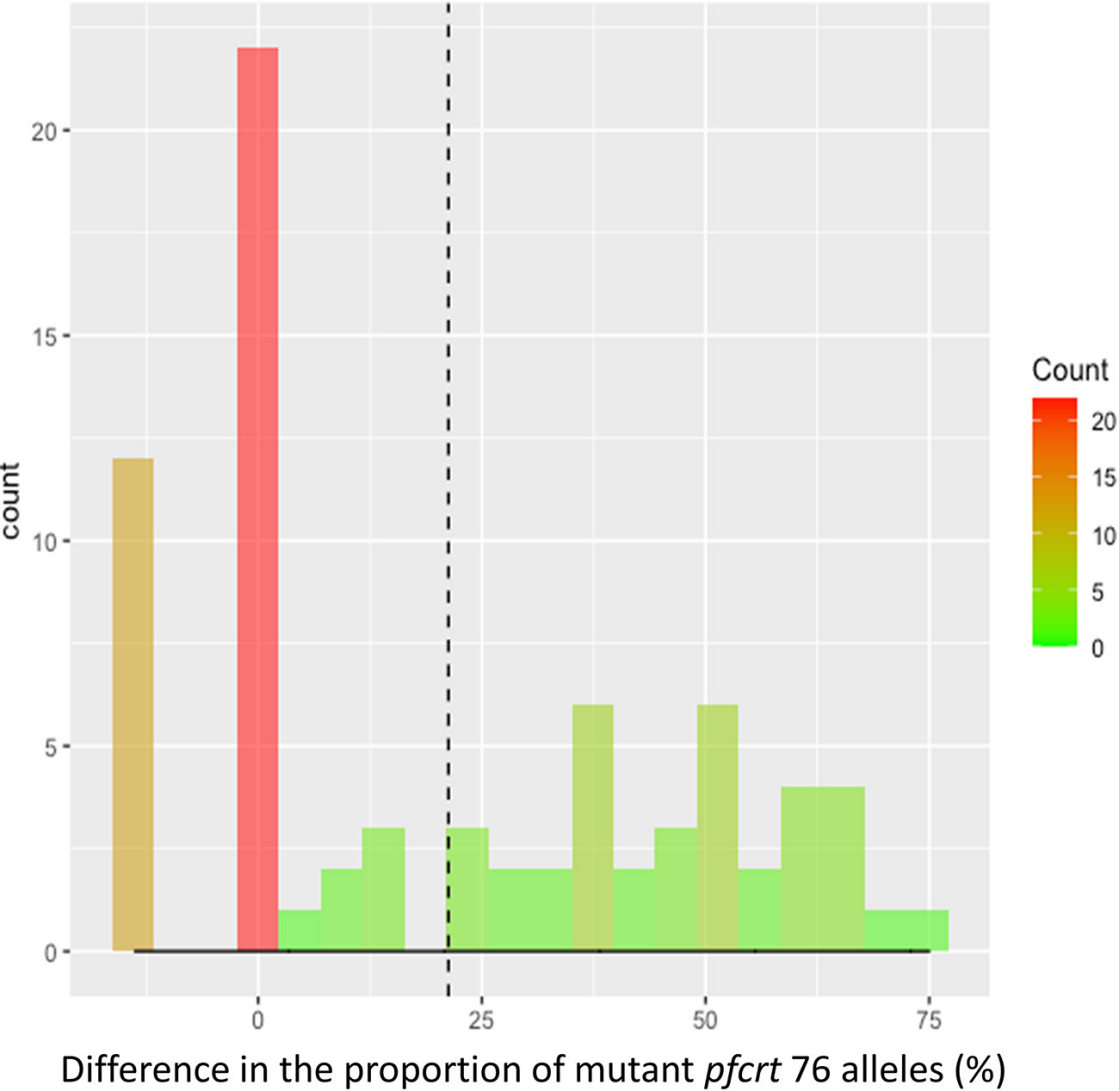
Distribution of the increase in the frequency of parasites with mutant *pfcrt* 76 alleles between the gametocyte and sporozoite stages for experimental infections where the proportion of parasites harboring the considered allele did not exceed 20% at the gametocyte stage. The comparison of the proportion of mutant *pfcrt* 76 alleles was restricted to experimental feedings where gametocyte samples had a proportion of mutant *pfcrt* alleles below 20%, defining these parasites as a minority variant. The difference of proportions between the gametocyte and sporozoite stages was computed, where a positive value meant an increase in the proportion of parasites with the mutant *pfcrt* allele at the sporozoite stage. The dashed line represents the average of this increase.

Next, we restricted our analysis to experimental feedings where gametocyte samples had a proportion of mutant *pfcrt* alleles below 20%, defining these parasites as a minority variant. We computed the difference of proportions between the gametocyte and sporozoite stages, where a positive value meant an increase in the proportion of parasites with the mutant *pfcrt* allele at the sporozoite stage. Through a simple chi-square test, we showed that the proportion of parasites with the minority allele at the sporozoite stage was significantly higher than zero (mean = 40%, *P < *1e−16), suggesting that minority variants had a competitive advantage (Fig. 4). A similar pattern was observed for the wild-type minority allele (mean = 21%, *P = *3.5e−9) (Fig. S6).

### Contribution of parasitological factors influencing the advantage of the minority variant.

To quantify the contribution of each factor influencing the advantage of this minority variant, we designed a generalized linear model through a forward approach (increasing sequentially the complexity of the model to find the most-parsimonious one) based on an improvement of the Akaike information criterion (AIC). This model aims at explaining the difference between the proportions of parasites with the mutant *pfcrt* allele at the gametocyte and sporozoite stages according to gametocytemia, oocyst count, and allelic diversity. It turned out that the most-parsimonious model involved a positive relationship with gametocytemia (coefficient = 0.046, *P = *4.49e−6) and negative relationships with the oocyst count (coefficient = −0.331, *P = *0.016) and genetic diversity at the gametocyte stage (coefficient = −6.692, *P = *5.68e−6) (Table 1).

**TABLE 1:** Results of the most-parsimonious generalized linear model explaining the increase in the frequency of *pfcrt* 76 mutant alleles between the gametocyte and sporozoite stages^*a*^

Parameter	Coefficient	*P* value
Intercept	9.579	1.41e−4
Gametocytemia	0.046	4.49e−6
Oocyst count	−0.331	0.016
MOI	−6.692	5.68e−5

a The results of the most-parsimonious generalized linear model explaining the increase in the frequency of *pfcrt* 76 mutant alleles between the gametocyte and sporozoite stages where the percentage of parasites harboring *pfcrt* 76 mutant alleles does not exceed 20% at the gametocyte stage. MOI, multiplicity of infection in the gametocyte population.

### Advantage of the minority variant: a general phenomenon?

**FIG 5:**
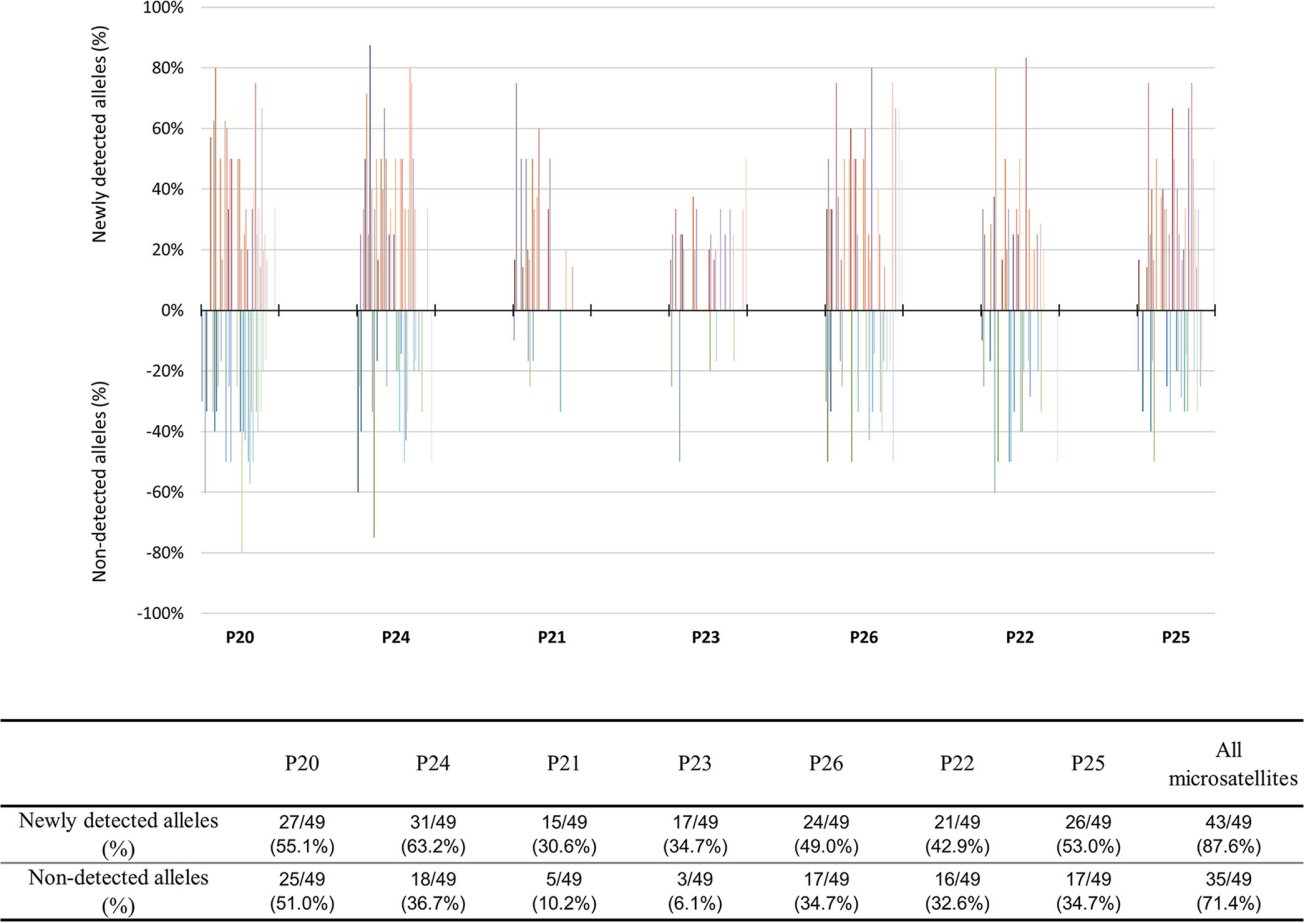
Frequencies of newly detected alleles and nondetected alleles in paired sporozoites for each feeding as a function of microsatellites (*x* axis). The allelic composition in sporozoite samples was compared to that in their paired gametocyte samples. Each bar represents the newly detected (frequency of >0%) or the nondetected (frequency of <0%) alleles in sporozoite samples for each paired gametocyte sample. The table shows, for each microsatellite, the frequency of newly detected or nondetected alleles in all sporozoite samples compared to their frequency in the paired gametocyte samples.

DNAs from P. falciparum-positive salivary glands and from gametocyte samples were successfully genotyped at 7 microsatellite loci for 49 blood donors and 560 salivary glands (Table S1). The multiplicity of infection (MOI) in the gametocyte samples was not different from that of the paired sporozoite bulks: the median MOI was 3 (mean = 3.47) for gametocyte samples versus 4 (mean = 3.96) for the sporozoite samples (Wilcoxon signed-rank test, *P = *0.120). At each microsatellite locus, the number of alleles varied from 4 to 16 (mean = 11.4) and was not different between gametocyte and sporozoite samples (chi-square test, *P = *0.998). Furthermore, the distribution of alleles at each microsatellite locus was not different (Table 2). All these data indicate that the allelic diversity between the gametocyte and sporozoite samples was not different at the metapopulation level. In contrast, when considering the blood donor level, we observed changes in allelic composition in sporozoite samples compared to their paired gametocyte samples (Fig. 5). We detected additional alleles in sporozoite samples for 43 of 49 feedings (87.6%), whereas for 35 feedings (71.4%), alleles identified in the gametocyte samples were not recovered in sporozoite samples. Details for each locus can be found in Fig. 5.

**TABLE 2:** Allele distribution in the gametocyte or sporozoite samples for each microsatellite locus

Microsatellite locus	Type of sample^*a*^	Number of observed alleles for the considered allele																		χ² test
P20	**Allele**	**132**	**139**	**142**	**145**	**147**	**150**	**153**	**156**	**159**	**162**	**165**	**168**	**171**	**174**	**177**	**180**	**183**	**186**	0.707
	Gameto	5	1	4	9	0	9	25	17	19	15	14	4	4	5	8	0	1	1	
	Sporo	4	2	6	5	2	7	26	19	19	15	10	5	5	6	12	1	0	0	

P24	**Allele**	**157**	**160**	**163**	**166**	**169**	**172**	**175**	**177**	**180**	**183**	**186**	**189**	192						0.359
	Gameto	0	15	25	25	18	9	8	5	8	8	3	4	1						
	Sporo	2	11	21	25	24	18	13	6	13	6	3	4	2						

P21	**Allele**	**65**	**68**	**71**	**74**	**77**	**80**	**83**	**86**	**92**										0.973
	Gameto	3	1	26	16	5	24	14	3	2										
	Sporo	3	3	31	19	5	29	17	5	2										

P23	**Allele**	**93**	**96**	**99**	**102**	**105**	**108**													0.780
	Gameto	6	21	42	3	0	0													
	Sporo	7	30	45	5	1	1													

P26	**Allele**	**159**	**162**	**165**	**168**	**171**	**174**	**177**	**180**	**183**	**186**	**189**	**192**	**198**	**201**	**213**				0.468
	Gameto	2	33	27	3	1	23	22	2	2	7	3	3	0	1	1				
	Sporo	3	33	32	3	3	26	26	4	2	8	5	3	3	2	0				

P22	**Allele**	**54**	**57**	**60**	**63**	**66**	**69**	**72**	**75**	**78**	**81**	**84**								0.671
	Gameto	0	1	11	9	29	21	13	17	3	6	0								
	Sporo	1	1	14	7	29	21	14	21	0	8	3								

P25	**Allele**	**93**	**96**	**99**	**102**	**105**	**108**	**111**	**114**	**117**										0.746
	Gameto	1	1	14	17	29	11	17	7	3										
	Sporo	6	5	10	19	36	13	17	11	5										

a Gameto, gametocyte samples; Sporo, sporozoite samples. The text in bold corresponds to all the alleles observed for the considered microsatellite locus.

## DISCUSSION

In this study, we investigated the transmission dynamics of malaria parasites through the mosquito vector from genetically diverse natural infections. Our results reveal that (i) the mosquito vector contributes to the spread of CQ-resistant parasites by maintaining subpatent genotypes and (ii) the minority genotype, whether wild-type or mutated, in the gametocyte population is favored during transmission through the vector.

Gametocyte carriers were identified in an area of high malaria transmission where infections are mostly asymptomatic and the complexity of infection is great (17, 18). In such infections, a high proportion of individuals carry gametocytes and contribute to parasite transmission (14, 19, 20). In this study, up to 10 coinfecting genotypes were identified in the gametocyte samples, which reflects high parasite exposure and a high level of acquired immunity (21, 22). Microscopy and even standard PCR do not allow the accurate determination of the relative densities of coinfecting genotypes, and this often represents a limitation in studies on transmission dynamics. Here, we identified microsatellite alleles in sporozoites that were not detected in gametocyte samples. These newly detected alleles were obviously already present in gametocytes but remained below the detection threshold of the quantitative PCR (qPCR). Even if we cannot preclude misamplification of low-density genotypes, our work confirms that gametocytes present at undetectable densities are infectious for the mosquito vector (17, 19).

Our data indicate that development within the mosquito favored minority, low-density genotypes, and this higher transmissibility of minority genotypes could represent a trade-off to maintain genetic diversity in the population (23, 24). A specific selection of genotypes by the mosquito cannot explain our results, as no differences in MOIs, allelic compositions, or allelic diversities were observed between gametocytes and sporozoites. Even if the microsatellite analysis is not able to formally demonstrate that low-density genotypes are favored by vectors, because the data produced are only qualitative (presence or absence of a given allele), these results strongly strengthen this hypothesis. As observed in the human host (25), multiple balances likely determine the outcome of malaria parasite infections in the vector. Indeed, we previously reported from natural P. falciparum infections that genetic complexity allows parasites to escape the mosquito’s immune responses (18) and that gametocytes are capable of sensing the genetic content within the infection to adapt inbreeding levels (17).

In Cameroon, chloroquine was used as a first-line treatment of uncomplicated malaria until 2002, when CQ resistance was reported throughout the country and the drug was deemed inefficient (26). Artemisinin-based combination therapies (ACTs) were adopted starting in 2004 and have been used nationwide since 2007. Molecular monitoring of CQ resistance reported the reemergence of wild-type parasites a few years after chloroquine removal; 45% of blood isolates carried the K76 allele in 2009 and 75% in 2012 (27). Here, the wild-type genotype was identified in 69% of the gametocyte samples. Our study may indicate stability in the prevalence of the wild-type genotype, but it would be necessary to genotype the asexual blood stages to further investigate this, as the genetic diversity might differ between the sexual and asexual populations (19). In any case, the increase in wild-type prevalence in Cameroon occurred to a lesser extent than in malaria-endemic countries in East Africa (3–6). Other ecological parameters determine the distribution of resistant and sensitive parasites, even if they are mostly linked to drug pressure. The prevalence of resistant genotypes decreases during the dry, low-transmission season, when drug pressure is limited (28, 29). CQ-resistant parasites are more frequent in rural areas, where self-medication is a common practice and chloroquine is still available from drug vendors (27, 30). Moreover, a higher prevalence of wild-type genotypes has been reported in clinical infections than in asymptomatic parasite carriers, which could be suggestive of higher virulence in wild-type parasites (27, 31). Alternatively, wild-type parasites are expected to have better fitness in more-immune individuals, such as asymptomatic infections in areas of high malaria transmission (32, 33). Nonetheless, the heterogeneity of the epidemiological context in different malaria settings renders predictions about the spread of drug selection difficult.

The role of mosquitoes in the transmission dynamics of drug-resistant genotypes has already been investigated (34, 35). In field studies among sympatric human and mosquito populations from Zambia, P. falciparum dihydrofolate reductase (*pfdhfr*) and *pfcrt*-resistant alleles were found at much lower frequencies in mosquitoes than in blood samples, and the authors suggested that the mosquito vector contributes to recovery of drug-sensitive parasites (34, 35). In our mosquito infection experiments, the frequencies of wild-type and mutant *pfcrt* alleles were similar over all gametocyte and sporozoite samples, which indicates that the cost of drug resistance observed in the human host does not occur in the mosquito vector. As we quantified the proportions of the *pfcrt* K76 allele in our samples, we further explored the dynamics of mutant and wild-type genotypes from gametocytes to sporozoites for each experiment and found that the least represented allele in the sexual stage population is more abundant in mosquito salivary glands, regardless of its identity, K76 or 76T. This result thus indicates a selective advantage of the minority allele in the mosquito vector. Negative frequency-dependent selection that maintains genetic diversity within populations has been largely described in the literature (36, 37). This pattern of selection would explain the persistence of drug-resistant genotypes in the absence of drug pressure in our study area, where malaria endemicity is high and genetic diversity great. Unfortunately, this may also have important epidemiological consequences, as it predicts the rapid reemergence and spread of resistant genotypes if antimalarial drugs that had previously selected resistant parasites are reintroduced for malaria prevention or treatment. Chloroquine has been massively used in Africa, including in Cameroon, since the coronavirus disease 2019 (COVID-19) pandemic (38), and thus, it will be important to follow the dynamics of the *pfcrt* K76T mutation and examine whether this novel change in drug pressure has induced an adaptive response by increasing the frequency of the *pfcrt* 76T allele.

The positive association we observed between gametocytemia and the increase in the minority *pfcrt* genotype strengthens the scenario of a competitive advantage of the minority allele in the mosquito vector. Conversely, the negative relationship between the MOI at the gametocyte stage and the increase in the minority *pfcrt* allele frequency suggests cooperative interactions, as more genotypes are at low densities in complex infections. Mixed infections lead to lower parasite burdens in mosquitoes (lower oocyst counts) but contribute to infecting more mosquitoes (higher infection prevalence), thereby increasing the chances of malaria parasite transmission to the next host (17, 18). Our results based on the *pfcrt* K76T mutation suggest that genetically diverse infections create a favorable environment for a genotype that would otherwise be less fit. We then provide evidence that heterogeneous interactions in mixed-genotype infections and the outcome of an infection depend on the interplay between competitive and cooperative behaviors.

Our study showed that the vector plays an important role in maintaining parasite genetic diversity and that the transmission of the minority *pfcrt* alleles by mosquitoes is possibly an evolutionary mechanism for the survival of P. falciparum genotypes with lower fitness in the human host. Further studies with a larger sample size are required to confirm our findings, and the genotyping of a broad set of polymorphisms using deep sequencing will be necessary to investigate whether the selective advantage of minority genotypes occurs. Indeed, our results may have important implications for malaria control, particularly in the persistence of resistant strains in the absence of drug pressure.

## MATERIALS AND METHODS

### Study sites and origin of samples.

The study was carried out over 2 years, from 2007 to 2008, during high-transmission (rainy) seasons. The recruitment sites were in primary schools from the Mfou district (3°43′N, 11°38′E), 26 km from the center of Yaoundé, Cameroon. This study was reviewed and approved by the Cameroonian National Ethics Committee (protocol number 039/CNE/MP/06). Naturally infected P. falciparum gametocyte carriers were identified by microscopy of a thick blood smear obtained by finger prick and stained in 10% Giemsa stain. Gametocyte densities were expressed as the number of gametocytes observed per 1,000 leukocytes, assuming a standard concentration of 8,000 leukocytes per microliter. Venous blood samples were drawn and used for mosquito feedings and gametocyte isolation. Children with trophozoite densities of >500 parasites per microliter were then treated with an artemisinin-based combination therapy according to national recommendations.

### Gametocyte isolation.

Gametocytes were isolated from 1 ml of serum-free blood using a MACS column (Miltenyi Biotec, Germany) as previously described (39). DNA from purified gametocytes was extracted with DNAzol (Molecular Research Center, Inc., USA) and was subjected to whole-genome amplification (WGA) using the GenomiPhi V2 DNA amplification kit (GE Health Care, Sweden). All DNA samples were frozen at −20°C until gametocyte genotyping.

### Experimental feedings.

For each mosquito feeding experiment, 2 batches of about 60 females of the local Ngousso laboratory strain of Anopheles coluzzii were fed with 400 μl of gametocyte-containing blood using the serum replacement procedure (18). Blood-fed mosquitoes were kept under standard insectary conditions (26°C, 80% relative humidity) with a daily 6% sucrose solution until dissections. At day 8 postinfection (p.i.), mosquitoes from 1 batch were dissected. The midguts were removed and stained in a 0.4% Mercurochrome solution, and the developed oocysts were counted by light microscopy. The prevalence of infection was defined as the proportion of infected mosquitoes among the total number of dissected mosquitoes and the infection intensity as the number of oocysts per P. falciparum-positive mosquito. At day 14 p.i., salivary glands from mosquitoes in the second batch were dissected and transferred into 200 μl of DNAzol. DNA extractions were performed according to the manufacturer’s protocol. A P. falciparum-specific PCR (PF1 5′-GGAATGTTATTGCTAACAC-3′ and PF2 5′-AATGAAGAGCTGTGTATC-3′) was carried out on salivary gland DNA to identify sporozoite-positive samples. DNA from positive salivary glands was frozen at −20°C until sporozoite genotyping.

### Genotyping of gametocyte and sporozoite samples.

Genotyping of the *pfcrt* 76 codon (chromosome 7) was performed on both gametocyte and sporozoite DNA with a real-time PCR assay using fluorescence resonance energy transfer (FRET) hybridization probes and a melting curve analysis as previously described (40). Each run included two control DNA samples of P. falciparum, i.e., the DNA from the CQ-susceptible F32/Tanzania strain corresponding to the *pfcrt* K76 wild-type allele and that of the CQ-resistant FCM29/Cameroon clone carrying the *pfcrt* 76T mutant allele.

DNA from gametocytes and DNA from positive salivary glands, after being pooled for each donor, were amplified to assess genetic polymorphism at 7 microsatellite loci located on 5 different chromosomes according to Anderson et al. (41) and Annan et al. (12). PCR products were resolved on an ABI Prism 3100 DNA genetic analyzer (Applied Biosystems, Foster City, CA, USA) using 500-LIZ as the internal size standard. Alleles were read under GeneMapper software (Applied Biosystems). The maximum number of alleles at the more polymorphic locus provided the minimum number of clones per isolate and determined the multiplicity of the infection.

### Quantification of mutant and wild-type alleles.

To assess the proportions of mutant and wild-type *pfcrt* 76 alleles in each sample, we used a hybridization probe-based FRET qPCR. We first produced standards with known proportions of mutant and wild-type *pfcrt* 76 alleles from laboratory strains (Fig. S1 and S2). All assays were performed in duplicate. The relative quantification of mutant/wild-type alleles of *pfcrt* 76 was determined using the plot of the negative first derivative of the fluorescence with respect to temperature (−*dF*/*dT*) versus temperature as previously described (42, 43). The relative amounts of mutant or wild-type alleles were determined by calculating the ratio of the melting peak heights of the high (mutant) or low (wild-type) melting temperature (*T_m_*) curves against the sum of the heights of both mutant and wild-type melting peaks, respectively, as follows: mutant % = [mutant *T_m_* peak height/(mutant *T_m_* peak height + wild-type *T_m_* peak height)] × 100, and wild-type % = [wild-type *T_m_* peak height/(mutant *T_m_* peak height + wild-type *T_m_* peak height)] × 100.

The proportions of *pfcrt* 76 alleles were assessed in field-derived gametocyte and sporozoite samples after validating the reproducibility of the quantification method (Fig. S1 and S2). Samples were tested only once due to the small amount of DNA and the number of assays to be performed.

### Statistical analysis.

Data that were not normally distributed were displayed as median values along with interquartile ranges and were compared using the Wilcoxon signed-rank test for two-group comparisons. Proportions were compared using the χ^2^ test or Fisher’s exact test, as appropriate. The relationship between the ratio of mutant to wild-type *pfcrt* 76 alleles and premixed standards was calculated by simple linear regression, and the strength of the relationship was assessed by correlation analysis using GraphPad Prism software (GraphPad, Inc., San Diego, CA, USA). Differences in comparisons were considered statistically significant if the *P* value was ≤0.05.

All the following statistical analyses were carried out using R (version 3.2) (44) and the lme4 package (45). To analyze the variations of proportions of *pfcrt* 76 alleles, we compared the proportion of wild-type *pfcrt* 76 alleles observed in the gametocyte samples to the proportion of wild-type *pfcrt* 76 alleles in the paired sporozoite samples, respectively. The same comparison was performed for the mutant *pfcrt* 76 alleles. The variations of proportions of the wild-type or mutant *pfcrt* 76 allele were analyzed using a logistic regression that took into account the donor effect because data were not independent (1 gametocyte sample corresponded to *n* sporozoite samples). The generalized linear mixed model fit by maximum likelihood (Laplace approximation) and the binomial family used the following formula: variations of proportions of *pfcrt* 76 alleles ∼ 1 + percentage in gametocyte sample + (1 | gametocyte sample).

The dynamics of *pfcrt 76* alleles was assessed by considering infections where an allele, either mutant or wild type, was present below the threshold of 20% in the gametocyte sample, i.e., it was considered a “minority” variant. Differences in the proportions of the minority parasite among the two developmental stages were checked using the chi-square test. The increase in the proportion of the minority variant was modeled through a generalized linear model in which the infection characteristics (gametocytemia, MOI, and oocyst count) were included as explanatory variables. We then used the function glm to identify the most-parsimonious model through a forward approach and test for significant interactions between explanatory variables and the minority allele increase.
