# Supplementary material for: The Rare, the Best: Spread of Antimalarial-Resistant Plasmodium falciparum Parasites by Anopheles Mosquito Vectors
Source: Microbiol Spectr. 2021 Oct 20;9(2):e00852-21. doi: 10.1128/Spectrum.00852-21 (PMC8528099; doi:10.1128/Spectrum.00852-21)

SUPPLEMETARY DATA

Appendix Table S1: Global data concerning the gametocyte, oocyst, and sporozoite samples

Gametocyte Carrier	Blood samples (Gametocytes)				Gut samples (Oocysts)			Salivary gland samples (Sporozoites)				
	Gametocyte density*	MOI†	% *pfcrt* 76 alleles		N‡	Mean§	Prevalence¶	N#	MOI‖	N**	Mean % *pfcrt* 76 alleles	
			K76	*76T*							*K76*	*76T*
**gc1**	23	NA	31.3	68.7	30	0.2	13.3	NA	NA	16	51.0	49.0
**gc3**	88	NA	0.0	100.0	45	3.8	42.2	NA	NA	11	41.7	58.3
**gc4**	11	NA	0.0	100.0	28	0.0	0.0	NA	NA	3	56.3	43.7
**gc6**	24	NA	100.0	0.0	23	0.5	21.7	NA	NA	24	57.7	42.3
**gc7**	901	2	0.0	100.0	45	40.3	48.9	9	2	24	45.2	54.8
**gc8**	17	NA	32.1	67.9	60	4.2	51.7	NA	NA	16	30.8	69.2
**gc9**	107	NA	21.1	78.9	48	2.0	29.2	NA	NA	17	28.9	71.1
**gc10**	177	NA	0.0	100.0	29	17.5	69.0	NA	NA	12	13.6	86.4
**gc11**	33	NA	49.5	50.5	50	3.4	40.0	NA	NA	4	49.6	50.4
**gc12**	18	NA	0.0	100.0	46	0.9	21.7	NA	NA	5	14.9	85.1
**gc13**	31	3	0.0	100.0	39	6.0	51.3	4	3	23	29.5	70.5
**gc14**	16	3	32.1	67.9	47	1.9	34.0	3	3	15	27.1	72.9
**gc16**	91	NA	58.7	41.3	19	2.1	42.1	NA	NA	17	43.9	56.1
**gc17**	154	3	0.0	100.0	37	2.5	10.8	3	3	15	0.0	100.0
**gc19**	95	NA	0.0	100.0	31	74.9	87.1	NA	NA	12	24.7	75.3
**gc20**	83	NA	76.3	23.7	49	45.9	65.3	NA	NA	19	55.8	44.2
**gc21**	20	NA	21.3	78.9	79	2.3	60.8	NA	NA	11	16.8	83.2
**gc22**	23	NA	47.3	52.7	36	3.1	61.1	NA	NA	17	47.6	52.4
**gc24**	23	4	34.1	65.9	32	3.4	68.8	7	4	18	47.3	52.7

**gc25**	154	1	0.0	100.0	26	43.6	80.8	3	3	8	10.1	89.9
**gc26**	2304	1	0.0	100.0	38	185.1	81.5	6	2	3	28.9	71.1
**gc27**	11	NA	0.0	100.0	24	0.0	0.0	NA	NA	10	38.9	61.1
**gc28**	21	NA	0.0	100.0	25	5.5	56.0	NA	NA	6	21.6	78.4
**gc35**	36	5	34.9	65.1	47	6.2	85.1	6	5	4	45.4	54.6
**gc36**	18	NA	74.4	25.6	45	5.8	91.1	NA	NA	3	7.2	92.8
**gc41**	144	NA	22.2	77.8	46	5.3	56.5	NA	NA	5	31.2	68.8
**gc47**	245	2	74.7	25.3	41	6.3	58.5	11	5	10	97.1	2.9
**gc48**	49	2	0.0	100.0	32	2.3	75.0	19	5	7	0.0	100.0
**gc49**	524	5	20.4	79.6	48	15.7	80.9	5	3	5	14.0	86.0
**gc50**	21	4	65.9	34.1	74	3.5	70.3	34	6	10	18.3	81.7
**gc52**	55	3	NA	NA	43	1.4	53.5	5	3	NA	NA	NA
**gc53**	64	5	NA	NA	21	7.1	76.2	3	4	NA	NA	NA
**gc56**	173	3	75.5	24.5	65	13.9	81.5	15	4	5	91.8	8.2
**gc60**	46	2	100.0	0.0	50	4.0	84.0	15	5	10	84.4	15.6
**gc63**	57	5	75.6	24.4	54	8.3	90.7	10	4	9	78.6	21.4
**gc64**	647	1	100.0	0.0	23	53.7	78.3	12	3	10	96.1	3.9
**gc66**	296	1	0.0	100.0	51	36.4	84.3	15	4	17	1.4	98.6
**gc67**	165	6	31.8	68.2	33	20.2	93.9	12	7	9	38.0	62.0
**gc68**	56	2	29.2	70.8	28	9.6	82.1	16	5	10	34.3	65.7
**gc69**	103	2	59.0	41.0	38	1.8	52.6	30	8	13	48.7	59.3
**gc70**	48	3	100.0	0.0	46	1.2	52.2	19	7	8	63.9	36.1
**gc71**	62	1	0.0	100.0	49	3.0	71.4	3	1	4	0.0	100.0
**gc72**	33	2	0.0	100.0	35	2.3	48.6	13	3	8	87.5	12.5
**gc76**	266	2	56.5	43.5	50	14.4	84.0	28	4	11	67.0	33.0
**gc78**	23	4	66.1	33.9	50	5.9	90.0	10	4	9	56.5	43.5
**gc79**	32	2	0.0	100.0	23	5.1	91.3	9	3	9	32.0	68.0
**gc80**	33	7	50.4	40.6	45	10.5	93.3	8	3	18	0.0	100.0

**gc81**	19	6	72.0	28.0	40	1.8	60.0	14	7	10	34.0	66.0
**gc83**	370	6	56.5	43.5	54	26.0	88.9	6	6	6	56.8	43.2
**gc85**	107	3	68.3	31.7	30	21.2	83.3	12	4	10	62.8	37.2
**gc86**	53	5	35.2	64.8	69	23.9	95.7	12	5	12	29.9	70.1
**gc88**	44	6	28.7	71.3	38	3.0	79.0	12	6	19	49.6	50.4
**gc89**	29	4	40.2	59.8	34	2.8	73.5	7	3	7	7.4	92.6
**gc90**	68	1	0.0	100.0	64	3.2	81.3	12	2	14	0.0	100.0
**gc93**	257	1	100.0	0.0	27	44.6	96.3	10	1	6	75.0	25.0
**gc95**	23	6	37.0	63.0	57	3.0	75.4	12	6	10	9.9	90.1
**gc96**	98	6	29.0	71.0	70	8.8	78.6	12	3	11	0.0	100.0
**gc97**	13	10	75.7	24.3	47	0.4	31.9	12	6	10	7.0	93.0
**gc99**	48	5	86.2	13.8	24	1.1	45.8	12	3	18	88.0	12.0
**gc102**	39	4	0.0	100.0	58	0.6	36.2	12	4	10	77.0	23.0
**gc105**	219	2	100.0	0.0	44	55.9	88.6	12	2	15	100.0	0.0
**gc106**	257	2	100.0	0.0	36	35.4	94.4	12	1	16	100.0	0.0
**gc108**	122	5	0.0	100.0	40	2.5	67.5	12	5	20	81.5	18.5
**gc111**	74	1	NA	NA	36	5.4	83.3	10	2	NA	NA	NA
**gc112**	47	7	100.0	0.0	41	8.5	85.4	12	6	9	100.0	0.0
**gc113**	40	1	0.0	100.0	48	2.2	77.1	12	2	10	96.1	3.9
**gc115**	49	3	0.0	100.0	44	3.6	70.5	12	4	10	3.0	97.0

NA: not available

* Gametocyte density was expressed as the number of gametocytes per microliter

† Multiplicity of the infection (MOI) in the gametocyte population

‡ Number of gut samples analyzed for oocyst content and prevalence of infection

§ Mean number of oocysts per gut sample

¶ Prevalence of infected mosquitoes

Number of salivary gland samples analysed for microsatellite profile

‖ Multiplicity of the infection (MOI) in sporozoite population

**Number of salivary gland samples analysed for *pfcrt* 76 genotyping

Appendix Figure S1: Quantification of mutant and wild-type *pfcrt* 76 alleles using FRET-based DNA hybridization probes. DNA from the FcM29/Cameroon clone carrying mutant *pfcrt* 76T allele was used as mutant control, whereas DNA from the F32/Tanzania strain carrying wild-type *pfcrt* K76 allele was used as wild-type control. We produced standards with a known proportion of mutant and wild-type *pfcrt* 76 alleles to quantify the proportion of *pfcrt* 76 alleles through the real-time PCR method and validate the reproducibility of the quantification method. Numbers 1–3 represent a F32/Tanzania: FcM29/Cameroon ratio of 7.5:2.5, 5:5, and 2.5:7.5, respectively. The figure shows the negative first derivative of fluorescence with respect to the temperature (-dF/dT) plotted against the temperature for the different mixtures of mutant and wild-type *pfcrt* 76 alleles. The two melting peaks of the mutant (*pfcrt* 76T) and the wild-type (*pfcrt* K76) alleles are clearly separated by ~5°C. In the mixtures of wild-type and mutant alleles, two peaks are obtained according to the proportion of each allele. The relative quantification of mutant or wild-type alleles was determined by calculating the ratio of melting peak heights of the high (mutant) or low (wild-type) temperature melting curves against the sum of heights of both mutant and wild-type melting peaks, respectively (22, 23):

$$\text{Mutant \%} = \frac{\text{Mutant Tm peak height}}{\text{Mutant Tm peak height} + \text{wild}-\text{type Tm peak height}} \text{ x } 100$$

$$\text{Wild-type \%} = \frac{\text{Wild}-\text{typeTm peak height}}{\text{Mutant Tm peak height} + \text{wild}-\text{type Tm peak height}} \text{ x } 100$$

These mixed samples (1-3) tested in duplicate showed the good reproducibility of the quantification method.

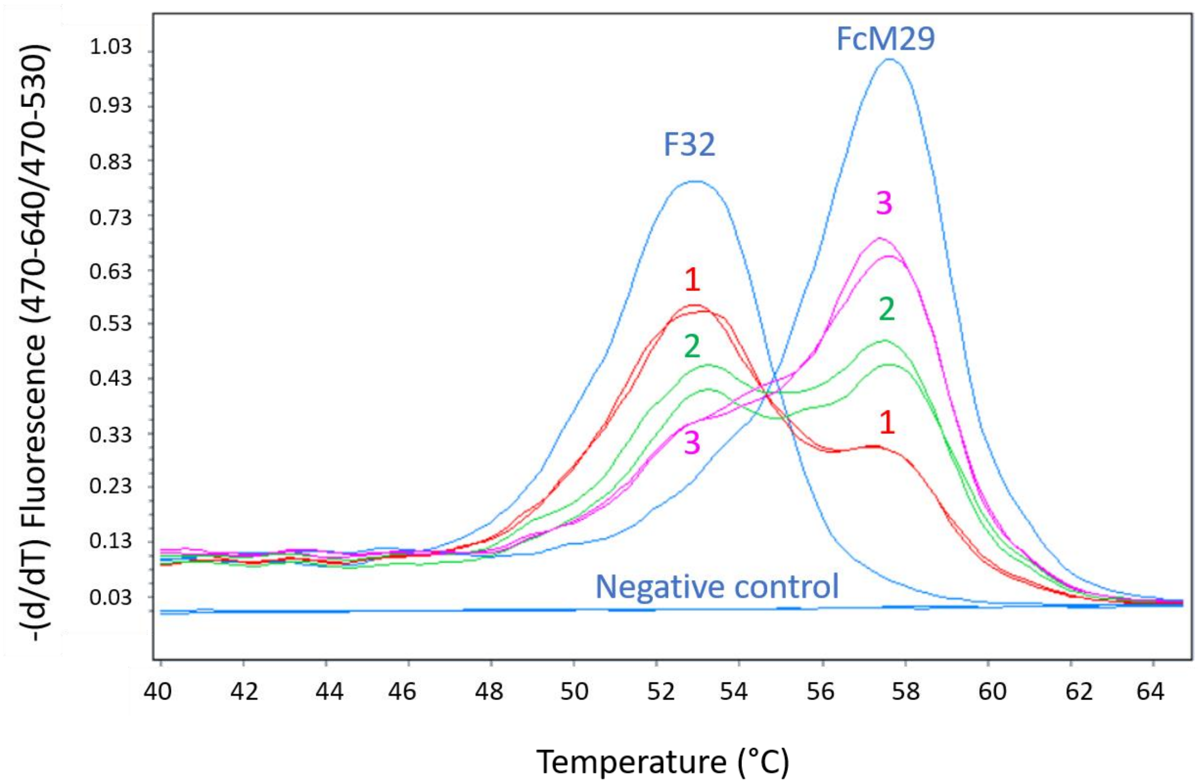
-(d/dT) Fluorescence (470-640/470-530)
1.03
0.93
0.83
0.73
0.63
0.53
0.43
0.33
0.23
0.13
0.03
FcM29
F32
3
1
2
2
3
1
Negative control
40
42
44
46
48
50
52
54
56
58
60
62
64
Temperature (°C)

Appendix Figure S2: Validation of the method for the quantification of the proportion of mutant *pfcrt* 76T and wild-type *pfcrt* K76 alleles.

The relative amount of mutant and wild-type *pfcrt* 76 alleles was calculated for each sample as described in the material and method section and plotted against the theoretical amount of mutant and wild-type alleles present in the pre-mixed standards. A standard curve was plotted using the pre-mixed DNA standards in the 0–100% range and the calculated proportion of wild-type (A) or mutant (B) alleles present in samples. Linear regressions provided good fitting curves (R^2 = 0.9831 and R^2 = 0.9834 for the wild-type allele and for the mutant allele, respectively), validating the method for the accurate quantification of the mutant and wild-type *pfcrt* 76 alleles in biological samples.

(A)

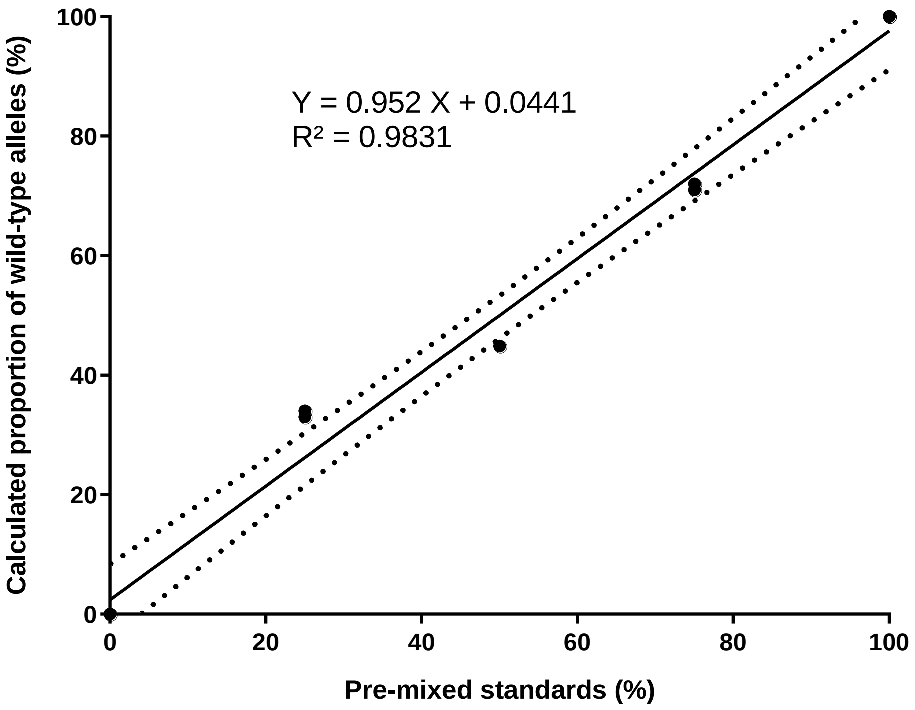

(B)

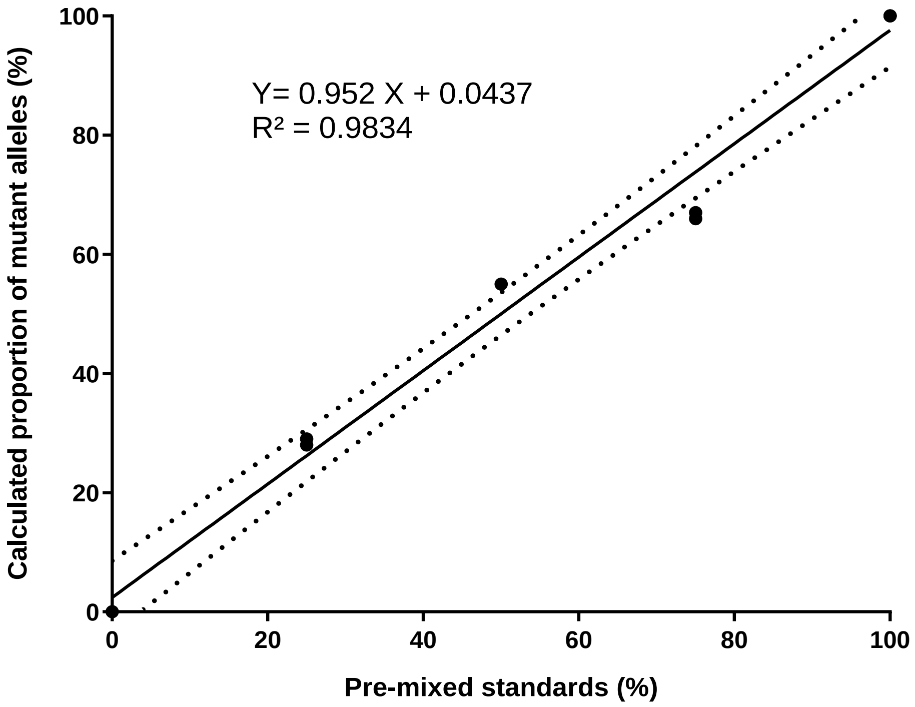
Y= 0.952 X + 0.0437
R² = 0.9834
Calculated proportion of mutant alleles (%)
Pre-mixed standards (%)
0
20
40
60
80
100

Appendix Figure S3: Box plot of wild-type *pfcrt* 76 alleles in gametocyte and sporozoite samples. The dots represent the observed proportion of wild-type *pfcrt* 76 alleles in the gametocyte samples and the observed average proportion of wild-type *pfcrt* 76 alleles in the sporozoite samples. Box plots represent the median, 25th and 75th percentiles, and the minimum and the maximum.

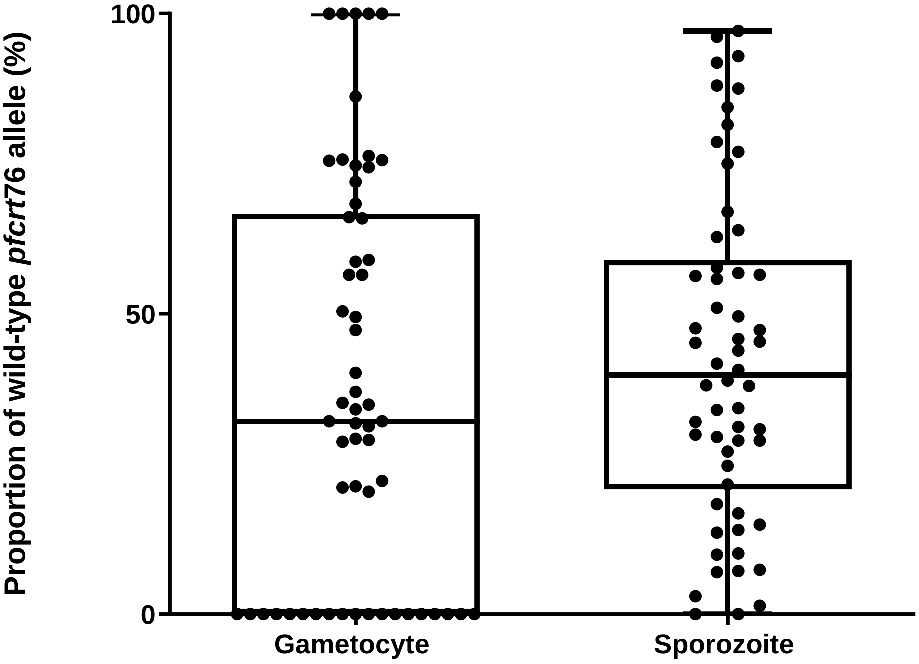

Appendix Figure S4: Dispersion of the proportion of wild-type *pfcrt* 76 alleles in gametocyte samples and in paired sporozoite samples. The dots represent the observed proportion of wild-type *pfcrt* 76 alleles in each sporozoite sample against the observed proportion of wild-type *pfcrt* 76 alleles in the paired gametocyte sample. The blue line represents the expected and theoretical distribution of dots if the vector has no influence in the transmission of *pfcrt* 76 alleles. The dot size is weighted by the number of samples

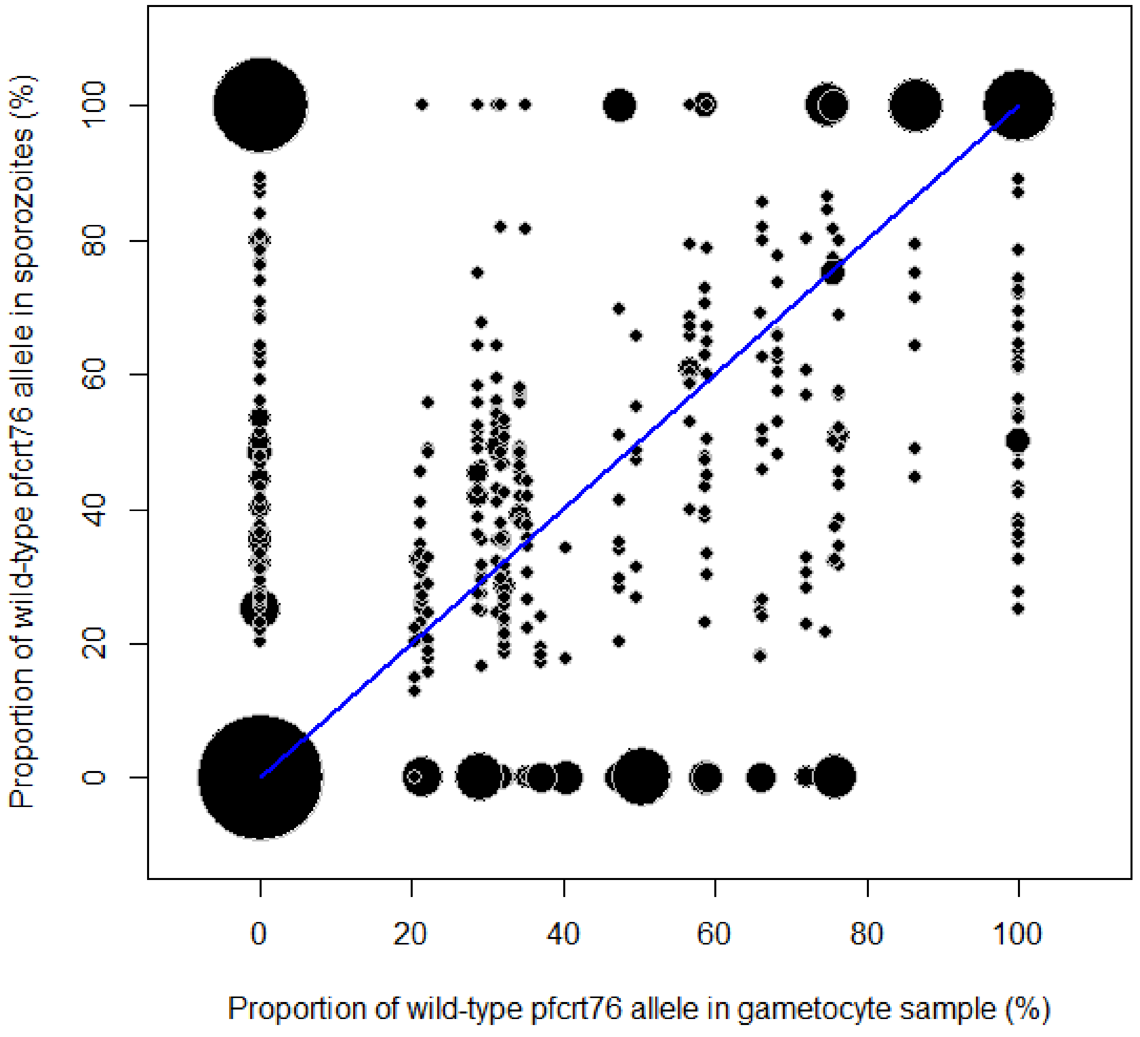

Appendix Figure S5: Logistic regression of the probability of a higher proportion of wild-type *pfcrt* 76 alleles in sporozoite samples as a function of the proportion of wild-type *pfcrt* 76 alleles in gametocyte samples. The proportion of wild-type *pfcrt* 76 alleles in sporozoite samples was compared to that in the paired gametocyte samples. The probability of having a higher proportion of wild-type *pfcrt* 76 alleles in sporozoite samples was calculated for each gametocyte sample using a logistic regression; the donor effect was taken into account. This probability (expressed in percentage) as a function of the proportion of wild-type *pfcrt* 76 alleles in gametocyte samples was plotted (red line). The blue line represents the expected probability if the variation of the proportion of wild-type *pfcrt* 76 alleles in sporozoite samples is randomly distributed (50% of a higher proportion and 50% of a lower proportion).

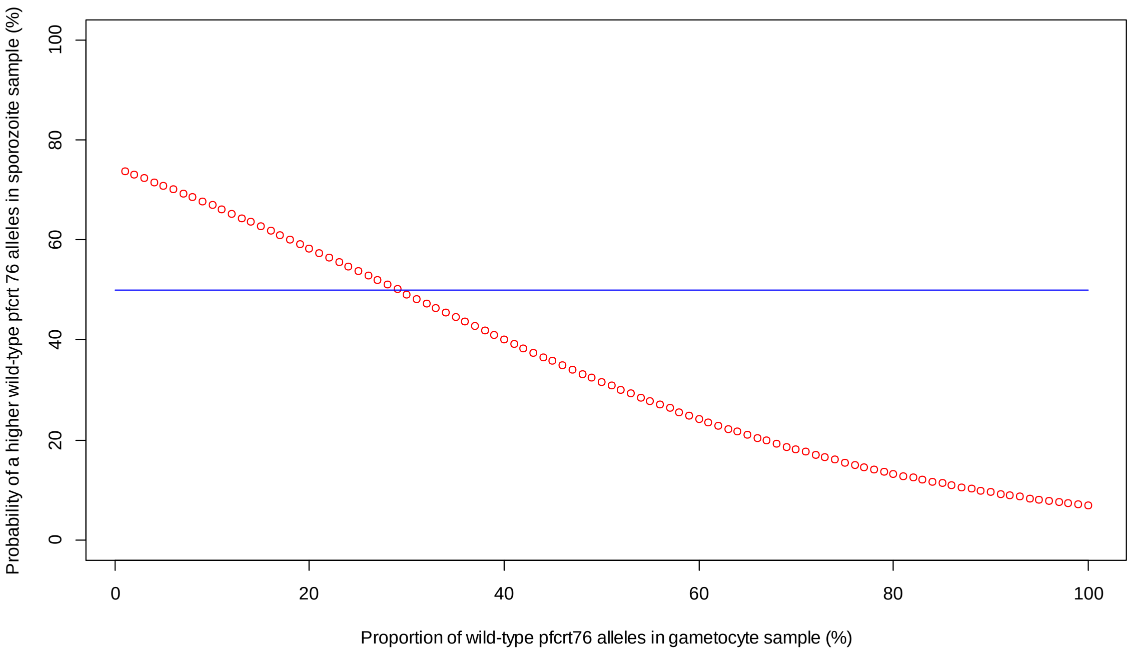

Appendix Figure S6: Distribution of the increase in the frequency of parasites with wild-type *pfcrt* 76 alleles between the gametocyte and sporozoite stages for experimental infections where the proportion of parasites harboring the considered allele does not exceed 20% at the gametocyte stage. Dashed line represents the average of this increase.

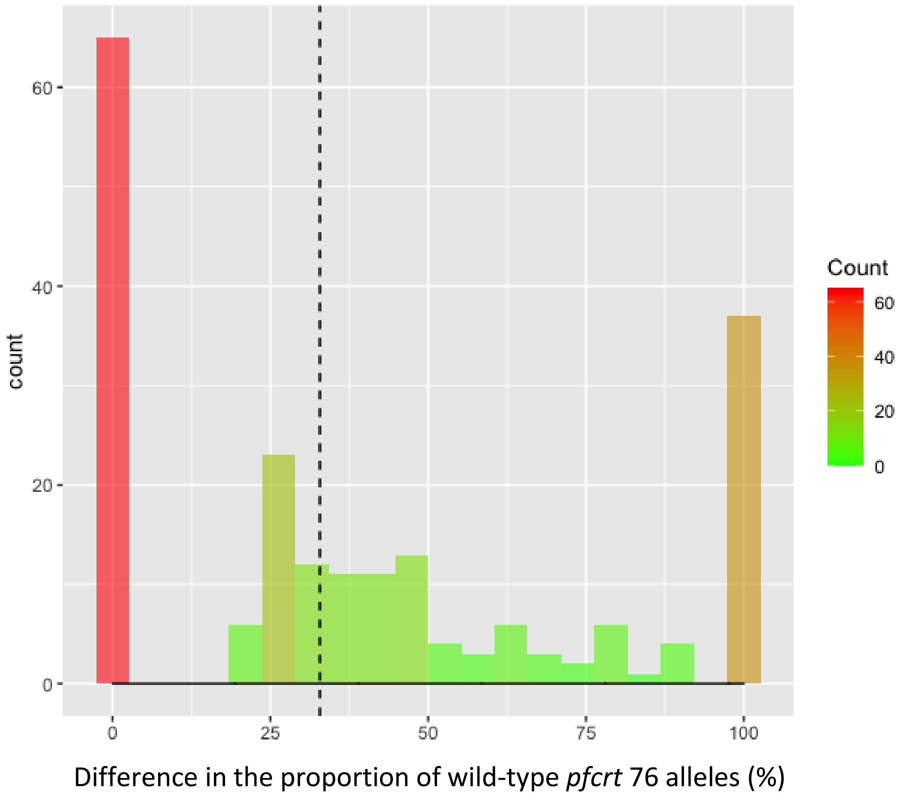

Supplement: SUPPLEMENTAL FILE 1 — Supplemental material. Download SPECTRUM00852-21_Supp_1_seq9.pdf, PDF file, 0.9 MB [file spectrum00852-21_supp_1_seq9.pdf]
